# Cognitive Predictors of Internalizing Symptoms in Clinically Anxious Youth

**DOI:** 10.1007/s10608-025-10612-2

**Published:** 2025-05-02

**Authors:** Dania Y. Amarneh, Michael J. Zvolensky, Eric A. Storch, Andres G. Viana

**Affiliations:** 1 University of Houston, Houston, USA; 2 The University of Texas MD Anderson Cancer Center, Houston, USA; 3 Baylor College of Medicine, Houston, USA; 4 Texas A&M University, College Station, USA; 5 Texas A&M Health Science Center, College Station, USA

**Keywords:** Temperament, Negative emotionality, Anxiety sensitivity, Interpretation biases, Internalizing symptoms

## Abstract

**Background:**

Internalizing disorders are the most common psychiatric disorders in youth and are associated with a host of deleterious outcomes (e.g., self-harm, substance use, interpersonal difficulties), highlighting the critical need for identifying risk factors that confer risk for these disorders. The present study investigated the unique and shared roles of two prominent cognitive biases—anxiety sensitivity and interpretation biases—as predictors of internalizing symptom severity in clinically anxious youth, above and beyond the effects of negative emotionality and after accounting for sociodemographic covariates.

**Method:**

A diverse sample of clinically anxious youth (*N* = 105; M_age_ = 10.09 years, SD = 1.22; 56.7% female; 49% ethnic minority) completed a diagnostic interview and self-report measures of interpretation biases, anxiety sensitivity, and internalizing symptom severity.

**Results:**

Hierarchical regression analyses revealed that both anxiety sensitivity (*b* = 0.77, 95% CI [0.53, 1.00], *sr*^*2*^ = 0.11) and interpretation biases (*b* = 0.21, 95% CI [0.11, 0.30], *sr*^*2*^ = 0.05) accounted for an additional 22% of unique variance in internalizing symptom severity (*p* <.001), above and beyond the effects of negative emotionality. Post hoc exploratory analyses identified the disease and social concerns facets of anxiety sensitivity, and the overgeneralization facet of interpretation biases, as predictors of internalizing symptoms.

**Conclusion:**

Future research should examine whether targeting cognitive biases would be beneficial among temperamentally labile youth.

## Introduction

Internalizing problems, such as anxiety and depression, are a common form of psychopathology among youth (Conley et al., 2023; Silva et al., 2020) with a lifetime prevalence ranging from 25–31% (Silva et al., 2020). Internalizing problems can emerge as young as three years old (Donohue et al., 2019; Luby, 2013) but show a dramatic increase in adolescence (Lunetti et al., 2022; Maciejewski et al., 2017; Nivard et al., 2017). This prevalence is particularly troubling given established associations between internalizing disorders and numerous adverse outcomes such as interpersonal difficulties, academic underachievement, self-harm, and substance use (Bai et al., 2020; Conley et al., 2023; Vergunst et al., 2023). Consequently, a better understanding of the risk factors that contribute to internalizing psychopathology is needed to inform the field about which youth may be most at risk (Beauchaine et al., 2008). To this end, the primary objective of this investigation was to examine the unique contributions of two cognitive risk factors—interpretation biases and anxiety sensitivity—to internalizing psychopathology, beyond the influence of temperamental negative emotionality, in a diverse sample of clinically anxious youth.

Negative emotionality refers to a child’s dispositional tendency to frequently experience negative emotions such as sadness, irritability, and fear (Davis et al., 2015; Lunetti et al., 2022; Snyder et al., 2015). Youth with high temperamental negative emotionality also display avoidance behaviors commonly seen among youth with internalizing disorders (American Psychiatric Association, 2022; Dodd et al., 2017). Past research supports strong associations between temperamental negative emotionality in childhood and internalizing symptoms and disorders (Crawford et al., 2011; Dodd et al., 2017; Lunetti et al., 2022; MacNeill & Pérez-Edgar, 2020; Snyder et al., 2015). Yet, given that temperamental dimensions are relatively stable over the course of early life through early adolescence (Kopala–Sibley et al., 2018; Shiner, 2015), negative emotionality may not be an ideal target for intervention, making it crucial to pinpoint additional modifiable underlying factors contributing to internalizing disorders.

Cognitive-behavioral theories posit that distorted, yet malleable, cognitive processes play a key role in the development, maintenance, and treatment of internalizing psychopathology (Bernaras et al., 2019; Epkins, 2000; Morea & Calvete, 2021; Rozenman et al., 2014; Weems et al., 2001). A particularly relevant cognitive distortion is interpretation biases (Cannon & Weems, 2010; Prinstein et al., 2005), defined as the tendency to interpret ambiguous or neutral stimuli negatively (Cannon & Weems, 2010). For example, a child exhibiting elevated levels of interpretation biases may perceive classmates whispering as negative talk about them, while a child without such biases might simply view it as a discussion of a different topic. Interpretation biases are associated with greater internalizing symptoms both cross-sectionally (Cannon & Weems, 2010; Raines et al., 2019; Trent et al., 2020) and longitudinally (Prinstein et al., 2005). Importantly, they are considered a malleable treatment target (Hallion et al., 2024; Vrijsen et al., 2024) through cognitive-bias modification training (Biagianti et al., 2020) or as part of CBT protocols (Micco et al., 2014; Pereira et al., 2018; Reuland & Teachman, 2014). However, most studies of interpretation biases among youth have examined these biases in isolation, neglecting the well-established covariation among different cognitive errors in youth with internalizing difficulties (Klein et al., 2019; Prinstein et al., 2005; Raines et al., 2019; Viana et al., 2017; Weems & Watts, 2005).

Anxiety sensitivity, defined as the fear of anxiety-related sensations due to the belief that they signal impending catastrophe (Reiss, 1991; Reiss et al., 1986; Taylor et al., 2007), is another cognitive bias strongly associated with youth internalizing psychopathology. Anxiety sensitivity can be conceptualized as a type of interpretation bias, yet focused more directly on the interoceptive sensations that characterize the experience of anxiety symptoms (Reiss, 1991). Anxiety sensitivity may involve cognitive concerns (e.g., fear of ‘going crazy’ when experiencing concentration difficulties), social concerns (e.g., fear that others will notice that they are feeling anxious), or physical concerns (e.g., fear of quickened heart rate; Taylor et al., 2007). Previous cross-sectional and longitudinal work has found that heightened anxiety sensitivity contributes to internalizing symptom severity in youth (Hernandez Rodriguez et al., 2020; Naragon-Gainey, 2010; Weems et al., 2002; Zavos et al., 2010). Statistically significant associations between anxiety sensitivity and interpretation biases in terms of internalizing symptoms have also been reported, although this research has largely focused on adults (Viana & Gratz, 2012).

Taken together, investigations on interpretation biases and anxiety sensitivity have enhanced our understanding of their role in youth internalizing symptoms (Ehrenreich-May et al., 2017; Pereira et al., 2018; Reuland & Teachman, 2014; Sherman et al., 2019), yet a test that examines the shared and unique effects of these factors within a single, comprehensive model has yet to be conducted. Advancing this knowledge is important because despite their shared variance (~ 30%; Viana & Gratz, 2012), past work suggests that interpretation biases and anxiety sensitivity are distinct and provide incremental value when evaluated concurrently (Olthuis et al., 2012). Specifically, interpretation biases are primarily comprised of cognitive distortions related to external stimuli (e.g., social interactions) while anxiety sensitivity is a measure of catastrophizing of internal states (e.g., elevated heart rate). While both reflect maladaptive cognitions, they differ in their stimulus focus (external vs. internal) and specific content domains (social/environmental threat vs. interoceptive threat). Thus, given the differences in the content of each cognitive bias (Haynes & Lench, 2003), examining their shared and unique contributions, above and beyond temperamental risk, is empirically and clinically relevant (Garb, 2003; Haynes & Lench, 2003; Smith et al., 2003).

Specifically, while it is evident that malleable cognitive biases such as interpretation biases and anxiety sensitivity (Ehrenreich-May et al., 2017; Pereira et al., 2018; Reuland & Teachman, 2014; Sherman et al., 2019) contribute to our understanding of internalizing symptoms in youth, their impact beyond temperamental constructs like negative emotionality is less clear. In fact, previous work in this area has typically focused on either cognitive-affective factors (e.g., Epkins, 2000; Jandrić et al., 2021; Schweizer et al., 2020) or biologically based factors (e.g., Fox et al., 2005; McDermott et al., 2009; Morales et al., 2022). Weaving together the study of biological vulnerabilities and cognitive risk factors can facilitate a clearer understanding of potential developmental trajectories of psychopathology (Hinshaw, 2017). For instance, there is accumulating evidence that temperament in early life may influence the development of cognitive processes such as attentional (Pérez-Edgar et al., 2014) and interpretation biases (Fox & Helfinstein, 2013; Henderson, 2010). While only a few investigations have examined temperamental and cognitive factors concurrently in relation to youth internalizing symptoms, they have yielded encouraging results (Pérez-Edgar et al., 2011; Smith et al., 2018; Viana et al., 2017). Clinically, identifying malleable risk factors is also necessary in order to better tailor treatments and improve outcomes for youth (Nye et al., 2023). Likewise, understanding these associations in preadolescence, before the critical developmental period where rates of internalizing disorders increase rapidly (Nivard et al., 2017), remains a critical next step.

The present study examined the predictive^1^ effects of interpretation biases and anxiety sensitivity on internalizing symptoms—above and beyond negative emotionality—in a diverse sample of clinically anxious youth. In line with the National Institute of Mental Health’s Research Domain Criteria (RDoC) initiative and its focus on dimensional approaches to psychopathology (Kozak & Cuthbert, 2016), a total score of internalizing symptoms was examined as the outcome in the current study as opposed to traditional categorical clusters (i.e., anxiety or depression). The focus on internalizing symptoms broadly maps onto the negative valence domain of the RDoC framework, which is characterized by behaviors that serve to mitigate external or interoceptive perceptions of harm, threat, or loss (Kozak & Cuthbert, 2016). Moreover, given the well-documented comorbidity of internalizing disorders and symptoms among youth with anxiety disorders (Franco et al., 2007; Kalin, 2020; Melton et al., 2016), this approach increases the generalizability and utility of our results. We hypothesized that negative emotionality, interpretation biases, and anxiety sensitivity would be positively associated with internalizing symptoms (Smith et al., 2018). We also expected that interpretation biases and anxiety sensitivity would account for a statistically significant amount of variance in internalizing symptoms, above and beyond the effect of negative emotionality. Given the broader nature of interpretation biases (vs. anxiety sensitivity), we also hypothesized that interpretation biases would account for a greater amount of variance in internalizing symptoms than anxiety sensitivity. Post hoc analyses were conducted to explore the effects of facets of both interpretation biases and anxiety sensitivity on internalizing symptoms. Age, sex, and race were evaluated as covariates given previous work identifying higher rates of internalizing symptoms in girls and marginalized racial/ethnic minorities (Conley et al., 2023; Nivard et al., 2017; Silva et al., 2020).

## Method

### Participants

The present study is a secondary data analysis utilizing data from a previously completed parent study investigating the role of maternal interpretation biases on child anxiety. Families were included if (a) the child was between the ages of 8–12 years, (b) the child had a current primary anxiety disorder (by either mother or child report), (c) the mother reported clinically significant levels of anxiety during the clinical interview or on the Depression, Anxiety, and Stress Scales (DASS; Lovibond & Lovibond, 1995), and (d) the child currently lived with the mother. Children were excluded from the study based on the following criteria: (a) physical disability impairing ability to use a computer, (b) borderline or extremely low intellectual functioning, (c) below average reading comprehension, (d) concurrent primary diagnosis of any non-anxiety disorder, (e) currently receiving psychological or pharmacological treatment for anxiety, (f) danger to self/others, and (g) non-English speaking child or parent.

A total of 152 mother–child dyads were initially enrolled and completed the first session in the parent study. Based on review of eligibility, 38 families were excluded for not meeting inclusion criteria (e.g., low child cognitive functioning, child not having an anxiety disorder according to clinical interview). Of the remaining dyads (n = 114), nine were excluded due to incomplete data or drop-out, leaving a final sample of 105 dyads who completed both sessions. Only child data was included in the current investigation.

The final sample in the present study was comprised of 105 children with anxiety disorders between 8 to 12 years of age (*M*_*age*_ = 10.07 years, *SD* = 1.22; 57% female). The sample was ethnically diverse; children self-identified as 39% White, 28.6% Hispanic, 16.2% mixed ethnicity, 14.3% African-American, and 1.9% Asian. Results of a semi-structured interview with research staff indicated that the most common diagnosis among youth participants was Generalized Anxiety Disorder (46.7%), followed by Social Anxiety Disorder (27.6%), Specific Phobias (16.2%), Separation Anxiety Disorder (7.6%), and Other Anxiety Disorders (1.9%). Over half of the sample (69.5%) had comorbid diagnoses, the most common being Specific Phobias (14.3%), Attention Deficit/Hyperactivity Disorder (12.4%), Generalized Anxiety Disorder (8.6%), Oppositional Defiant Disorder (7.6%), Major Depressive Disorder (7.6%), Separation Anxiety Disorder (5.7%), and Social Anxiety Disorder (5.7%).

### Procedure

All study procedures were approved by the University of Houston's Institutional Review Board. Anxious mothers and children were recruited for participation in the parent study through local advertisements and community events. Upon indicating interest in the study, research staff provided a description of the study and administered a brief screen via telephone to assess for inclusion and exclusion criteria. Informed consent and assent were obtained from the mother and child prior to all study procedures. Across two study visits scheduled one week apart, mothers and children separately completed semi-structured clinical interviews with trained research staff as well as self-report measures of the constructs of interest. Families were debriefed at the end of their participation, and provided with the results of the diagnostic evaluation, relevant evidence-based recommendations, and contact information for local treatment providers. Additionally, participants were compensated $50 per session, and children were able to select a small toy after both sessions.

### Measures

#### MINI International Neuropsychiatric Interview for Children and Adolescents (MINI-KID; Sheehan et al., 1998, 2010)

The MINI-KID is a semi-structured diagnostic interview for children ages 6 to 17 years. It assesses for the presence of common psychiatric diagnoses through discrete modules, per DSM-5 criteria (American Psychiatric Association, 2013). Each module consists of screening items; if the screening items are endorsed, the assessor proceeds through the full module to determine diagnostic criteria. All items require yes/no responses, and assessors may ask additional questions for clarification. Two separate assessments were conducted—one with the child, and one with the mother responding to questions about the child. MINI-KID interviews were conducted by either the principal investigator or an advanced graduate student under the PI’s supervision. Graduate student assessors were trained to use the MINI-KID by observing videotaped samples of interviews conducted by the principal investigator. Assessors were required to meet perfect inter-rater reliability with the PI in three interviews before conducting two independent interviews supervised by the PI. All MINI-KID interviews were videotaped and all diagnoses made were reviewed during supervision sessions with assessors. No instances of diagnostic disagreement were found.

#### Early Adolescent Temperament Questionnaire, Short Form (EATQ; Ellis & Rothbart, 2001).

The EATQ is a 65-item, child-completed questionnaire designed to assess temperamental traits in late childhood through late adolescence. The measure yields 11 temperament subscales (i.e., activation control, activity level, affiliation, attention, fear, frustration, high intensity pleasure, inhibitory control, perceptual sensitivity, pleasure sensitivity, and shyness) and two behavioral scales (i.e., aggression and depressive mood). Items are rated on a 5-point Likert scale, ranging from 1 (*almost never true*) to 5 (*almost always true*). Subscale scores are obtained by averaging responses to items of each subscale (i.e., subscale scores range from 1–5). The negative emotionality (α = 0.72) component of temperament used in the present study was calculated by aggregating the means of the aggression, fear, frustration, shyness, and depressed mood subscales (Snyder et al., 2015). Although previous work is mixed on the cross-cultural invariance of the measure, the negative emotionality facet in particular has demonstrated strong construct validity and invariance across racial and ethnic groups (de Boo & Kolk, 2007; Lawson et al., 2021; Snyder et al., 2015).

#### Children’s Negative Cognitive Error Questionnaire (CNCEQ; Leitenberg et al., 1986).

The CNCEQ is a 24-item self-report measure that assesses the degree to which children interpret ambiguous events in an overly negative manner. The CNCEQ yields a total cognitive distortion score and four subscales (i.e., catastrophizing, α = 0.77; overgeneralization, α = 0.78; personalizing, α = 0.78; selective abstraction, α = 0.74). Each item consists of a hypothetical vignette followed by a negative interpretation of the vignette. The child is asked to rate on a 5-point Likert scale the degree to which they would interpret the situation in the same way (1 = *not at all like I would think* to 5 = *almost exactly like I would think*). Past work has documented good test–retest reliability, internal consistency, and construct validity across racial and ethnic minority groups for the CNCEQ scores (Leitenberg et al., 1986; Marsee et al., 2008; Pössel et al., 2024). In this study, the CNCEQ total score (α = 0.93) was used as a self-report measure of interpretation biases. Additionally, each subscale score was used as a predictor in post hoc exploratory analyses.

#### Childhood Anxiety Sensitivity Index (CASI; Silverman et al.,^1991^, ^2003^)

The CASI is an 18-item self-report questionnaire that assesses the fear of anxiety-related sensations in children and adolescents. Participants are asked to rate the extent to which they experience fearful reactions to normal anxiety responses on a 3-point scale (1 = *none*, 2 = *some*, 3 = *a lot*). A total score for anxiety sensitivity is obtained by summing the responses to each item, with scores ranging from 18 to 54. The measure also yields four subscales assessing fear of anxiety-related sensations in the following domains: disease concerns (e.g. “It scares me when my heart beats fast;” α = 0.84), social concerns (e.g. “I don’t want others to know when I’m afraid;” α = 0.56), mental incapacitation concerns (e.g. “When I am afraid, I fear I may be going crazy;” α = 0.64), and unsteady concerns (e.g. “It scares me when I have trouble catching my breath;” α = 0.72; Silverman et al., 2003). Evidence for the reliability and validity of the measure across racial and ethnic groups is mixed (Francis et al., 2019a, 2019b; Isolan et al., 2012; Martin et al., 2016). In this study, the CASI total score was used as a predictor in the main model and demonstrated excellent internal consistency (α = 0.90). The four subscales were examined as predictors in post hoc exploratory analyses.

#### Revised Child Anxiety and Depression Scale (RCADS; Chorpita et al., 2000)

The RCADS is a self-report measure designed to assess symptom severity of the following internalizing disorders in children, per DSM-IV-TR criteria (American Psychiatric Association, 2000): Separation Anxiety Disorder, Social Anxiety Disorder, Panic Disorder, Obsessive Compulsive Disorder, Generalized Anxiety Disorder, and Major Depressive Disorder. The child is asked to respond to 47 questions on a Likert scale (0 = *never* to 3 = *always*) regarding the extent to which they agree with statements indicative of anxiety (e.g., “I worry that bad things will happen to me”) and depression (e.g., “nothing is much fun anymore”). The RCADS has shown good internal consistency in both clinical and community samples of youth (Chorpita et al., 2000), as well as acceptable measurement invariance across racial/ethnic groups (Stevanovic et al., 2017; Trent et al., 2013). The RCADS total score, which showed excellent internal consistency (α = 0.95) in the current sample, was used as an index of internalizing symptom severity (the criterion variable).

### Data Analytic Plan

Correlations, t-tests, and analyses of variance were conducted to examine main and interactive effects of demographic variables (i.e., sex, age, race) on internalizing symptoms, negative emotionality, anxiety sensitivity, and interpretation biases. Second, a series of hierarchical regression analyses were conducted to examine the variance in internalizing symptom severity (RCADS total score) explained by anxiety sensitivity (CASI total score) and interpretation biases (CNCEQ total score), above and beyond the effects of negative emotionality (EATQ negative emotionality score). In Step 1 of the model, sociodemographic covariates were entered. In Step 2, the EATQ negative emotionality score was entered. Finally, in Step 3, the CASI and CNCEQ total scores were entered. The total scores of both cognitive biases were entered in a separate step, subsequent to negative emotionality, to examine both their unique and cumulative incremental contributions, respectively.

Post hoc exploratory analyses were conducted to examine the unique variance accounted for by the facets of the CASI and CNCEQ, respectively. The first of these regression models included sociodemographic covariates in Step 1, the EATQ negative emotionality score and the total score of the CNCEQ in Step 2, and the four CASI subscales (i.e. disease concerns, unsteady concerns, mental incapacitation concerns, and social concerns) in Step 3. In the last model, the CASI total score replaced the CNCEQ total score in Step 2, and the CNCEQ subscales (i.e., catastrophizing, overgeneralization, personalizing, and selective abstraction) were entered in Step 3. All analyses were conducted in SPSS version 29.0.

## Results

### Preliminary Analyses

There were no missing data across any of the study variables. Examination of collinearity diagnostics did not suggest excess multicollinearity as all VIF values were below 5.0 and tolerance values were greater than 0.2 (Kim, 2019); collinearity statistics are included for each variable in the regression tables. Distributions for all study variables approximated normality (skewness <|2.25|; George & Mallery, 2003; kurtosis <|1.96|; George & Mallery, 2010). Bivariate correlations for all study variables are presented in Table 1.

**Table 1:** Bivariate Correlations (*N* = 105)

	1	2	3	4	5	6	7	8	9	10	11	12	13
1. Age	–												
2. Negative Emotionality	.01	–											
3. Anxiety Sensitivity	−.11	.65**	–										
4. Interpretation Biases	.16	.64**	.56**	–									
5. Internalizing Symptoms	−.02	.72**	.78**	.71**	–								
6. CASI: Disease Concerns	−.13	.54**	.93**	.50**	.72**	–							
7. CASI: Unsteady Concerns	−.02	.61**	.85**	.53**	.63**	.70**	–						
8. CASI: Mental Incapacitation Concerns	−.04	.42**	.74**	.37**	.54**	.64**	.58**	–					
9. CASI: Social Concerns	−.12	.58**	.67**	.40**	.60**	.49**	.47**	.33**	–				
10. CNCEQ: Catastrophizing	.14	.58**	.58**	.90**	.65**	.53**	.52**	.38**	.40**	–			
11. CNCEQ: Overgeneralization	.11	.55**	.42**	.90**	.64**	.38**	.42**	.26**	.29**	.74**	–		
12. CNCEQ: Personalization	.12	.58**	.50**	.89**	.62**	.46**	.46**	.30**	.37**	.74**	.72**	–	
13. CNCEQ: Selective Abstraction	.21*	.69**	.51**	.90**	.62**	.41**	.49**	.40**	.38**	.74**	.75**	.74**	–
Mean	10.07	2.62	28.10	48.45	45.80	11.72	6.58	3.93	5.83	12.30	12.15	12.11	11.89
Standard Deviation	1.22	0.57	7.29	18.05	12.17	3.67	2.13	1.28	1.67	5.27	5.14	4.90	4.81
Range	8–12	1.31–4.14	18–49	24–102	27.19–81.22	8–23	4–12	3–8	3–9	6–29	6–30	6–26	6–24

Age, Demographics Questionnaire; Negative Emotionality, Early Adolescent Temperament Questionnaire, Short Form (EATQ; Ellis & Rothbart, 2001); Anxiety Sensitivity, Disease Concerns, Unsteady Concerns, Mental Incapacitation Concerns, Social Concerns, Childhood Anxiety Sensitivity Index (CASI; Silverman et al., 1991); Interpretation Biases, Catastrophizing, Overgeneralization, Personalization, Selective Abstraction, Children’s Negative Cognitive Error Questionnaire (CNCEQ; Leitenberg et al., 1986); Internalizing Symptoms, Revised Child Anxiety and Depression Scale (RCADS; Chorpita et al., 2000)

^*^p <.05; ** *p* <.01; *** *p* <.001

Child age demonstrated a statistically significant association with CNCEQ selective abstraction scores and was therefore included as a covariate in the model examining the CNCEQ subscales as predictors. In terms of child sex differences, females endorsed statistically significantly higher scores than males in CASI unsteady concerns (Female *M* = 7.03, *SD* = 2.31; Male *M* = 5.98, *SD* = 1.73; *t* [103] = -2.58; *p* = 0.011) and CNCEQ catastrophizing (Female *M* = 13.60, *SD* = 5.60; Male *M* = 10.58, *SD* = 4.27; *t* [102.96] = − 3.14; *p* = 0.002). Child sex was therefore included as a covariate in both post hoc regression models. There were no racial differences among the study variables’ total scores, (*p*s ranging from 0.179 to 0.743). As a result, race was not included as a covariate in any of the regression analyses. Finally, negative emotionality, anxiety sensitivity (and its subscales), interpretation biases (and its subscales), and internalizing symptoms were all statistically significantly and positively associated with one another (*r*s ranging from 0.26 to 0.93; Table 1).

### Hierarchical Regression Analysis

A summary of the main hierarchical regression analysis using the CASI and CNCEQ total scores as predictors of internalizing symptoms is presented in Table 2. Given the lack of statistically significant associations with the assessed sociodemographic covariates, none were included in this model. The overall model was statistically significant as the predictor variables altogether explained 73.1% of the overall variance in internalizing symptom severity (*F* [3, 101] = 91.50, *p* <. 001). Step 1 of the model, which included negative emotionality, was statistically significant, accounting for 52% of unique variance. Step 2 of the model, in which the total scores of both cognitive biases (i.e., anxiety sensitivity and interpretation biases) were entered, was also statistically significant, accounting for 22% of additional variance (*p* < 0.001). Anxiety sensitivity and interpretation biases were both statistically significant predictors of internalizing symptoms, with anxiety sensitivity accounting for over twice the variance explained by interpretation biases. Negative emotionality also remained statistically significant in Step 2.

**Table 2:** Hierarchical Regression Model Predicting Internalizing Symptom Severity Using CASI and CNCEQ Total Scores (*N* = 105)

	Δ*R*^2^	*b*	*SE*	*β*	*t*	*p*	*CI(l)*	*CI(u)*	*sr*^*2*^	*Tolerance*	*VIF*
Step 1	.52***										
Negative emotionality		15.37***	1.50	.72	10.47	<.001	12.46	18.29	.52	1.00	1.00
Step 2	.22***										
Negative emotionality		4.73**	1.63	.22	2.91	.004	1.50	7.96	.02	0.46	2.17
Anxiety sensitivity		0.77***	0.12	.46	6.54	<.001	0.53	1.00	.11	0.54	1.86
Interpretation biases		0.21***	0.05	.31	4.42	<.001	0.11	0.30	.05	0.55	1.81

Negative Emotionality, Early Adolescent Temperament Questionnaire, Short Form (EATQ; Ellis & Rothbart, 2001); Anxiety Sensitivity, Childhood Anxiety Sensitivity Index (CASI; Silverman et al., 1991); Interpretation Biases, Children’s Negative Cognitive Error Questionnaire (CNCEQ; Leitenberg et al., 1986); Internalizing Symptoms, Revised Child Anxiety and Depression Scale (RCADS; Chorpita et al., 2000)

^*^p <.05; ** *p* <.01; *** *p* <.001

### Post Hoc Exploratory Analyses

#### Model Using CASI Subscales as Predictors

A summary of the hierarchical regression analysis using the four CASI subscales as predictors is presented in Table 3. The overall model was statistically significant, accounting for 75.4% of variance in internalizing symptom severity (*F* [7, 97] = 42.37, *p* <. 001). Step 1 of this model, which included sex as a covariate, was not statistically significant (*R*^*2*^ = 0.001, *p* = 0.731). Step 2, which included negative emotionality and interpretation biases (i.e., CNCEQ total score), was statistically significant and explained 62% of unique variance (*p* < 0.001). Both negative emotionality and interpretation biases emerged as statistically significant predictors of internalizing symptoms. Finally, in Step 3, the four CASI subscales were entered as predictors. This step was statistically significant, accounting for 13% of additional variance in the model (*p* < 0.001). Only the disease concerns and social concerns subscales of the CASI emerged as predictors. Negative emotionality and interpretation biases remained statistically significant in this step.

**Table 3:** Hierarchical Regression Model Predicting Internalizing Symptom Severity Using CASI Subscales (*N* = 105)

	Δ*R*^2^	*b*	*SE*	*β*	*t*	*p*	*CI(l)*	*CI(u)*	*sr*^*2*^	*Tolerance*	*VIF*
Step 1	.00										
Sex		0.83	2.41	.03	0.35	.731	− 3.95	5.61	.00	1.00	1.00
Step 2	.62***										
Sex		− 2.24	1.51	−.09	− 1.48	.142	− 5.24	0.76	.01	0.97	1.03
Negative emotionality		9.71***	1.70	.45	5.71	<.001	6.33	13.08	.12	0.59	1.70
Interpretation biases		0.29***	0.05	.43	5.37	<.001	0.18	0.40	.11	0.58	1.72
Step 3	.13***										
Sex		− 2.46	1.29	−.10	− 1.90	.060	− 5.02	0.11	.01	0.91	1.10
Negative emotionality		4.75**	1.67	.22	2.84	.006	1.43	8.06	.02	0.42	2.40
Interpretation biases		0.22***	0.05	.33	4.76	<.001	0.13	0.31	.06	0.54	1.84
CASI Disease concerns		1.02***	0.27	.31	3.82	<.001	0.49	1.54	.04	0.40	2.52
CASI Unsteady concerns		0.05	0.46	.01	0.12	.907	− 0.86	0.96	.00	0.39	2.55
CASI Mental incapacitation concerns		0.82	0.64	.09	1.27	.206	− 0.46	2.09	.00	0.56	1.80
CASI Social concerns		1.17*	0.47	.16	2.50	.014	0.24	2.11	.02	0.61	1.63

Sex, Demographics Questionnaire; Negative Emotionality, Early Adolescent Temperament Questionnaire, Short Form (EATQ; Ellis & Rothbart, 2001); Anxiety Sensitivity, Disease Concerns, Unsteady Concerns, Mental Incapacitation Concerns, Social Concerns, Childhood Anxiety Sensitivity Index (CASI; Silverman et al., 1991); Interpretation Biases, Children’s Negative Cognitive Error Questionnaire (CNCEQ; Leitenberg et al., 1986); Internalizing Symptoms, Revised Child Anxiety and Depression Scale (RCADS; Chorpita et al., 2000)

^*^p <.05; ** *p* <.01; *** *p* <.001

#### Model Using CNCEQ Subscales as Predictors

A summary of the hierarchical regression analysis using the four CASI subscales as predictors is presented in Table 4. The overall model was statistically significant, accounting for 76.5% of variance in internalizing symptom severity (*F* [8, 96] = 38.96, *p* <. 001). Step 1 of this model, which included age and sex as covariates, was not statistically significant (*R*^*2*^ = 0.002, *p* = 0.926). Step 2, which included negative emotionality and anxiety sensitivity (i.e., CASI total score) was statistically significant and explained 69% of unique variance (*p* < 0.001). Both negative emotionality and anxiety sensitivity emerged as statistically significant predictors of internalizing symptoms. Finally, Step 3, in which the four CNCEQ subscales were entered, was also statistically significant, explaining 7% of additional variance in the model (*p* < 0.001). Only the CNCEQ overgeneralization subscale emerged as a statistically significant predictor. Negative emotionality and anxiety sensitivity remained statistically significant in this step, while sex emerged as a statistically significant predictor as well.

**Table 4:** Hierarchical Regression Model Predicting Internalizing Symptom Severity Using CNCEQ Subscales (*N* = 105)

	Δ*R*^2^	*b*	*SE*	*β*	*t*	*p*	*CI(l)*	*CI(u)*	*sr*^*2*^	*Tolerance*	*VIF*
Step 1	.00										
Age		− 0.19	1.00	−.02	− 0.19	.848	− 2.17	1.78	.00	0.98	1.02
Sex		0.89	2.44	.04	0.37	.716	− 3.95	5.73	.00	0.98	1.02
Step 2	.69***										
Age		0.56	0.57	.06	0.98	.329	− 0.57	1.68	.00	0.95	1.05
Sex		− 2.64	1.39	−.11	− 1.90	.060	− 5.40	0.12	.01	0.96	1.05
Negative emotionality		7.79***	1.58	.36	4.93	<.001	4.65	10.92	.07	0.57	1.77
Anxiety sensitivity		0.94***	0.13	.56	7.49	<.001	0.69	1.18	.17	0.55	1.82
Step 3	.07***										
Age		0.23	0.53	.02	0.43	.666	− 0.83	1.29	.00	0.82	1.22
Sex		− 3.72**	1.34	−.15	− 2.78	.007	− 6.37	− 1.06	.02	0.86	1.16
Negative emotionality		4.66**	1.57	.22	2.96	.004	1.54	7.78	.02	0.45	2.20
Anxiety sensitivity		0.85***	0.12	.51	7.06	<.001	0.61	1.09	.12	0.47	2.13
CNCEQ Catastrophizing		0.21	0.22	.09	0.95	.347	− 0.23	0.64	.00	0.27	3.68
CNCEQ Overgeneralization		0.75***	0.21	.32	3.64	<.001	0.34	1.15	.03	0.33	3.07
CNCEQ Personalization		− 0.01	0.22	−.01	− 0.07	.949	− 0.44	0.42	.00	0.32	3.10
CNCEQ Selective abstraction		− 0.16	0.23	−.06	− 0.69	.492	− 0.63	0.31	.00	0.29	3.48

Age, Sex, Demographics Questionnaire; Negative Emotionality, Early Adolescent Temperament Questionnaire, Short Form (EATQ; Ellis & Rothbart, 2001); Anxiety Sensitivity, Childhood Anxiety Sensitivity Index (CASI; Silverman et al., 1991); Interpretation Biases, Catastrophizing, Overgeneralization, Personalization, Selective Abstraction, Children’s Negative Cognitive Error Questionnaire (CNCEQ; Leitenberg et al., 1986); Internalizing Symptoms, Revised Child Anxiety and Depression Scale (RCADS; Chorpita et al., 2000)

^*^p <.05; ** *p* <.01; *** *p* <.001

## Discussion

The present study examined the role of two cognitive biases (i.e., interpretation biases and anxiety sensitivity) in explaining variance in internalizing symptoms, above and beyond the role of negative emotionality, in a diverse sample of clinically anxious youth. As hypothesized, all study variables were positively inter-correlated. This is consistent with extant literature reporting statistically significant associations between anxiety sensitivity, interpretation biases, negative emotionality, and internalizing symptoms in youth (Pérez-Edgar et al., 2011; Smith et al., 2018; Viana et al., 2017). In the main regression model, the hypothesis that interpretation biases and anxiety sensitivity would account for a statistically significant amount of variance, above and beyond the effect of negative emotionality, was supported. Specifically, both cognitive biases emerged as statistically significant predictors in the regression model. This finding is consistent with extant literature (e.g., Cannon & Weems, 2010; Hernandez Rodriguez et al., 2020; Raines et al., 2019) on the prominent role of these cognitive biases in predicting internalizing symptoms in youth. The results further underscore the notion that cognitive distortions can compound risk for internalizing symptoms in youth with temperamental vulnerabilities (e.g., negative emotionality). The findings also are in line with cognitive-behavioral theories of anxiety and depressive disorders which posit cognitive distortions as central to the etiology and maintenance of these conditions (Bernaras et al., 2019).

Contrary to our expectation that interpretation biases would account for a greater amount of unique variance in internalizing symptoms than anxiety sensitivity, the results of the hierarchical regression demonstrated that anxiety sensitivity accounted for over twice the variance explained by interpretation biases. This result may be attributed, in part, to the sample’s characteristics. Specifically, among clinically anxious (vs. depressed) youth, the degree of catastrophizing of interoceptive experiences (i.e., anxiety sensitivity) may be particularly relevant to their internalizing symptoms. This contrasts with the broader scope of interpretation biases, which do not specifically focus on internal sensations but instead reflect a general tendency to interpret situations negatively. Such bias may feature more prominently among youth with mood disorders. Indeed, one study conducted across two samples of clinic-referred and community-based youth found that interpretation biases were higher in youth with depression only, as opposed to only anxiety or comorbid diagnoses (Stevanovic et al., 2016).

Relatedly, and consistent with past work (Viana & Gratz, 2012), it is worth noting that interpretation biases and anxiety sensitivity shared 31% of variance in our sample, underscoring that these are related but distinct constructs. Our findings bolster the assertion that there is incremental utility in examining both of these cognitive biases concurrently (Olthuis et al., 2012). Indeed, in our main model, anxiety sensitivity and interpretation biases accounted for 11% and 5% of unique variance in internalizing symptoms, respectively, after accounting for negative emotionality. Therefore, while these constructs do share some similarities, the content by which their respective measures is comprised enough contributes unique information to our understanding of internalizing symptoms (Smith et al., 2003).

The results of the post hoc exploratory analysis examining the CASI subscales as predictors of internalizing symptoms revealed that only the disease concerns and social concerns facets of anxiety sensitivity predicted internalizing symptoms. This finding suggests that the fear of others noticing symptoms of anxiety in social settings, as well as associating physical symptoms such as racing heartbeat with impending physical illness, may be more relevant to internalizing symptoms than concentration difficulties and general physiological discomfort. Indeed, past studies have found that these two facets of anxiety sensitivity to be the most relevant subscales for clinically anxious youth (Silverman et al., 2003).

The diagnostic composition of the sample is also worth noting. In the present sample, the second most common primary diagnosis after generalized anxiety disorder was social anxiety disorder (SAD), with 26.7% meeting criteria for the disorder. Additionally, 5.7% of the sample met criteria for SAD as their secondary diagnosis. A diagnosis of SAD, characterized by fear of social situations where one might be exposed to scrutiny (American Psychiatric Association, 2022), is strongly related to the social concerns facet of anxiety sensitivity (Alkozei et al., 2014; Francis et al., 2019a, 2019b). Specifically, youth with SAD often report strong fears of the potential consequences of public displays of anxiety, which encourages avoidance and exacerbates the severity of the disorder (Miers et al., 2014). Silverman et al., (2003) further reported that in samples of clinically anxious youth, the highest levels of CASI social concerns were found among youth with SAD. Our findings regarding the CASI disease concerns facet are also consistent with previous work identifying this subscale as uniquely relevant to anxiety and panic symptoms (Leen-Feldner et al., 2005). Furthermore, this result provides additional empirical support for the notion that fearing physiological sensations, due to their potential to signal serious medical emergencies, is an important marker of internalizing psychopathology.

In the third regression model examining the predictive effects of the CNCEQ subscales, only the overgeneralization facet of interpretation biases emerged as a statistically significant predictor of internalizing symptoms. This finding suggests that overgeneralizing the results of limited experiences is particularly relevant to broadband internalizing symptoms, as has been reported previously (Cannon & Weems, 2010; Schwartz & Maric, 2015; Stevanovic et al., 2016; Tairi et al., 2016; Weems et al., 2001). Indeed, research attempting to specify whether overgeneralization errors are more relevant to anxiety versus depression has yielded mixed findings. For example, across community (*N* = 257) and clinical (*N* = 201) samples of youth, Stevanovic et al. (2016) found that while the CNCEQ overgeneralization subscale explained variance in both anxiety and depressive symptoms, it was most strongly related to depressive symptoms. Conversely, Tairi et al. (2016) found that overgeneralization was associated with anxiety but not depressive symptoms. Collectively, our findings and existing research suggest that overgeneralization errors may be best understood as a contributor to internalizing problems more broadly.

Study findings should be interpreted in light of its limitations. Although there is strong evidence that temperament (MacNeill & Pérez-Edgar, 2020; Shiner, 2015) and cognitive biases are a risk for (vs. a biproduct of) internalizing psychopathology (Creswell & O’Connor, 2011; Field & Lester, 2010), the cross-sectional nature of the data prevents drawing causal inferences regarding the direction of association. Future longitudinal investigations exploring the role of prior internalizing symptoms in the severity of current cognitive biases would further explicate the associations between these constructs*.* Additionally, the use of self-report measures to quantify symptom severity may have contributed to common method variance. That is, while a clinician-administered structured interview was used to derive diagnoses from both parent and child reports, the predictor and outcome variables used in the regression analyses were self-reported by the child participants. However, research has shown that when assessing internalizing symptoms and internal states (i.e., cognitive distortions), self-report measures may be preferable to parent-reports, given that child internal distress is often unnoticed or underreported by parents (Caqueo-Urízar et al., 2022). Furthermore, our findings may have been influenced by the item content of the RCADS. Specifically, the phrasing of items in the RCADS such as, “I feel worried when I think someone is angry with me,” may resonate more with a child exhibiting negative interpretation biases than with a child with heightened anxiety sensitivity. Conversely, youth with elevated anxiety sensitivity may be more likely to endorse items such as “I suddenly feel as if I can't breathe when there is no reason for this.” This may have influenced our tests of incremental validity by potentially amplifying the association of one cognitive bias over the other with the outcome measure. Thus, future work utilizing alternative measures of internalizing symptoms (e.g., teacher reports; behavioral observations) is warranted. Finally, the relatively low internal consistency of the CASI mental incapacitation and social concerns subscales (αs = 0.64 and 0.56, respectively), although comparable to those found in other studies (e.g., Amarneh et al., 2022; Hensley & Varela, 2008; Leen-Feldner et al., 2008), are a limitation. Specifically, Leen-Feldner et al.’s (2008) study yielded similar internal consistency estimates for the mental incapacitation and social concerns subscales (αs = 0.54 and 0.61, respectively) in a community-based sample of trauma-exposed youth, as did Hensley and Varela’s (2008) investigation in a sample of school-aged youth after exposure to Hurricane Katrina (αs = 0.66 and 0.50, respectively). The relatively low internal consistency of the social concerns subscale may be due, in part, to this subscale being comprised of only three items (vs. the 8-item disease subscale and the 4-item unsteady concerns subscale). Moreover, given that each of the three items assess distinct manifestations of social concerns (i.e., others noticing anxiety, the importance of maintaining control, and concealing emotions; Silverman et al., 2003), a low alpha can be considered acceptable (Taber, 2018).

Despite these limitations, the findings of this study retain clinical and empirical utility. Given the relative stability of temperamental facets such as negative emotionality (Shiner, 2015), the identification of interpretation biases and anxiety sensitivity as incrementally relevant in the development of internalizing symptoms can inform future studies of evidence-based clinical interventions. Specifically, available treatments that target interpretation biases and anxiety sensitivity are brief and effective (Hallion et al., 2024; Knapp et al., 2020; Sherman et al., 2019; Vrijsen et al., 2024). Focusing these treatments on reducing concerns regarding the social and physical consequences of anxiety, as well as overgeneralization errors, may be especially relevant. Interestingly, despite the stability of negative emotionality, extant literature on neuroticism suggests that intervening on negative emotionality may be worthwhile (Barlow et al., 2014; Sauer-Zavala et al., 2017). For example, CBT treatments targeting emotion dysregulation, such as the Unified Protocol for Children and Adolescents (Ehrenreich-May et al., 2017), result in reductions in neuroticism as well. This is thought to be a function of the significant overlap between characteristics of neuroticism/negative emotionality and internalizing symptoms (e.g., fear, sadness, irritability). As such, identifying youth who display negative emotionality through early assessment and utilizing intervention strategies to prevent the further development of internalizing symptoms may result in lessened functional impairment. Additionally, given the influence of temperamental traits in the development of cognitive biases (Fox & Helfinstein, 2013; Henderson, 2010), it may be valuable to examine negative emotionality as a moderator of the relation between cognitive biases and internalizing symptoms in a future investigation with a larger sample size to further elucidate this association and inform intervention research.

Overall, the present investigation found that, in a sample of clinically anxious youth, both interpretation biases and anxiety sensitivity predicted internalizing symptom severity above and beyond negative emotionality. Post hoc analyses also revealed that concerns regarding the social and physical consequences of anxiety sensations, as well as overgeneralization errors, were key predictors. Given that interpretation biases and anxiety sensitivity are targetable in treatment (e.g., Knapp et al., 2020; Vrijsen et al., 2024), these findings can inform future work on interventions for clinically anxious youth by highlighting two promising cognitive targets.

## Data Availability

No datasets were generated or analysed during the current study.

## Footnotes

1 The term and its variations (e.g., predicting) are used throughout the manuscript in a statistical sense, without suggesting a causal relationship between variables or constructs.

## References

[CR1] Alkozei, A., Cooper, P. J., & Creswell, C. (2014). Emotional reasoning and anxiety sensitivity: Associations with social anxiety disorder in childhood. *Journal of Affective Disorders,**152–154*, 219–228. 10.1016/j.jad.2013.09.014 24120086 10.1016/j.jad.2013.09.014 PMC3878593

[CR2] Amarneh, D. Y., Trent, E. S., Zvolensky, M. J., & Viana, A. G. (2022). The relationship between anxiety sensitivity and PTSD symptom severity among trauma-exposed inpatient adolescents. *Cognitive Therapy and Research*. 10.1007/s10608-022-10294-0

[CR3] American Psychiatric Association. (2000). DSM-IV-TR. *DSM Library*. 10.1176/appi.books.9780890420249.dsm-iv-tr

[CR4] American Psychiatric Association. (2013). *Diagnostic and statistical manual of mental disorders. (5th ed.)*. 10.1176/appi.books.9780890425596

[CR5] American Psychiatric Association. (2022). *Diagnostic And Statistical Manual Of Mental Disorders, Fifth Edition, Text Revision (DSM-5-TR)*. 10.1176/appi.books.9780890425787

[CR6] Bai, S., Ricketts, E. J., Thamrin, H., Piacentini, J., Albano, A. M., Compton, S. N., Ginsburg, G. S., Sakolsky, D., Keeton, C. P., Kendall, P. C., & Peris, T. S. (2020). Longitudinal study of sleep and internalizing problems in youth treated for pediatric anxiety disorders. *Research on Child and Adolescent Psychopathology,**48*(1), 67–77. 10.1007/s10802-019-00582-x 10.1007/s10802-019-00582-x PMC6925631 31506757

[CR7] Barlow, D. H., Sauer-Zavala, S., Carl, J. R., Bullis, J. R., & Ellard, K. K. (2014). The nature, diagnosis, and treatment of neuroticism: Back to the future. *Clinical Psychological Science,**2*(3), 344–365. 10.1177/2167702613505532

[CR8] Beauchaine, T. P., Neuhaus, E., Brenner, S. L., & Gatzke-Kopp, L. (2008). Ten good reasons to consider biological processes in prevention and intervention research. *Development and Psychopathology,**20*(3), 745–774. 10.1017/S0954579408000369 18606030 10.1017/S0954579408000369 PMC2690981

[CR9] Bernaras, E., Jaureguizar, J., & Garaigordobil, M. (2019). Child and adolescent depression: A review of theories, evaluation instruments, prevention programs, and treatments. *Frontiers in Psychology,**10*, 543. 10.3389/fpsyg.2019.00543 30949092 10.3389/fpsyg.2019.00543 PMC6435492

[CR10] Biagianti, B., Conelea, C., Brambilla, P., & Bernstein, G. (2020). A systematic review of treatments targeting cognitive biases in socially anxious adolescents. *Journal of Affective Disorders,**264*, 543–551. 10.1016/j.jad.2019.12.002 32056778 10.1016/j.jad.2019.12.002 PMC7024067

[CR11] Cannon, M. F., & Weems, C. F. (2010). Cognitive biases in childhood anxiety disorders: Do interpretive and judgment biases distinguish anxious youth from their non-anxious peers? *Journal of Anxiety Disorders,**24*(7), 751–758. 10.1016/j.janxdis.2010.05.008 20554426 10.1016/j.janxdis.2010.05.008 PMC2922394

[CR12] Caqueo-Urízar, A., Urzúa, A., Villalonga-Olives, E., Atencio-Quevedo, D., Irarrázaval, M., Flores, J., & Ramírez, C. (2022). Children’s mental health: Discrepancy between child self-reporting and parental reporting. *Behavioral Sciences,**12*(10), 401. 10.3390/bs12100401 36285970 10.3390/bs12100401 PMC9598658

[CR13] Chorpita, B. F., Yim, L., Moffitt, C., Umemoto, L. A., & Francis, S. E. (2000). Assessment of symptoms of DSM-IV anxiety and depression in children: A revised child anxiety and depression scale. *Behaviour Research and Therapy,**38*(8), 835–855. 10.1016/s0005-7967(99)00130-8 10937431 10.1016/s0005-7967(99)00130-8

[CR14] Conley, C. S., Hilt, L. M., & Gonzales, C. H. (2023). Internalizing in adolescents and young adults. In *APA handbook of adolescent and young adult development* (pp. 505–523). American Psychological Association. 10.1037/0000298-031

[CR15] Crawford, N. A., Schrock, M., & Woodruff-Borden, J. (2011). Child internalizing symptoms: Contributions of child temperament, maternal negative affect, and family functioning. *Child Psychiatry & Human Development,**42*(1), 53–64. 10.1007/s10578-010-0202-5 20734130 10.1007/s10578-010-0202-5

[CR16] Creswell, C., & O’Connor, T. G. (2011). Interpretation bias and anxiety in childhood: Stability, specificity and longitudinal associations. *Behavioural and Cognitive Psychotherapy,**39*(2), 191–204. 10.1017/S1352465810000494 20875165 10.1017/S1352465810000494

[CR17] Davis, S., Votruba-Drzal, E., & Silk, J. S. (2015). Trajectories of internalizing symptoms from early childhood to adolescence: Associations with temperament and parenting. *Social Development,**24*(3), 501–520. 10.1111/sode.12105

[CR18] de Boo, G. M., & Kolk, A. M. (2007). Ethnic and gender differences in temperament, and the relationship between temperament and Depressive and Aggressive mood. *Personality and Individual Differences,**43*(7), 1756–1766. 10.1016/j.paid.2007.05.012

[CR19] Dodd, H. F., Hudson, J. L., & Rapee, R. M. (2017). Temperament in Youth Internalizing Disorders. In *Treatments for Psychological Problems and Syndromes* (pp. 504–524). John Wiley & Sons, Ltd. 10.1002/9781118877142.ch31

[CR20] Donohue, M. R., Whalen, D. J., Gilbert, K. E., Hennefield, L., Barch, D. M., & Luby, J. (2019). Preschool depression: A diagnostic reality. *Current Psychiatry Reports,**21*(12), 128. 10.1007/s11920-019-1102-4 31748851 10.1007/s11920-019-1102-4 PMC7259424

[CR21] Ehrenreich-May, J., Kennedy, S. M., Sherman, J. A., Bilek, E. L., Buzzella, B. A., Bennett, S. M., & Barlow, D. H. (2017). *Unified Protocols for Transdiagnostic Treatment of Emotional Disorders in Children and Adolescents: Therapist Guide*. Oxford University Press.

[CR22] Ellis, L. K., & Rothbart, M. (2001). *Early Adolescent Temperament Questionnaire—Revised*. 10.1037/t07624-000

[CR23] Epkins, C. C. (2000). Cognitive specificity in internalizing and externalizing problems in community and clinic-referred children. *Journal of Clinical Child Psychology,**29*(2), 199–208. 10.1207/S15374424jccp2902_6 10802829 10.1207/S15374424jccp2902_6

[CR24] Field, A. P., & Lester, K. J. (2010). Is there room for ‘development’ in developmental models of information processing biases to threat in children and adolescents? *Clinical Child and Family Psychology Review,**13*(4), 315–332. 10.1007/s10567-010-0078-8 20811944 10.1007/s10567-010-0078-8

[CR25] Fox, N. A., & Helfinstein, S. M. (2013). The Contribution of Temperament to the Study of Social Cognition: Learning Whether the Glass Is Half Empty or Half Full. In M. R. Banaji & S. A. Gelman (Eds.), *Navigating the Social World: What Infants, Children, and Other Species Can Teach Us. *Oxford University Press. 10.1093/acprof:oso/9780199890712.003.0010

[CR26] Fox, N. A., Henderson, H. A., Marshall, P. J., Nichols, K. E., & Ghera, M. M. (2005). Behavioral inhibition: Linking biology and behavior within a developmental framework. *Annual Review of Psychology,**56*, 235–262. 10.1146/annurev.psych.55.090902.141532 15709935 10.1146/annurev.psych.55.090902.141532

[CR27] Francis, S. E., Manley, S., & Doyle, S. (2019a). A psychometric evaluation of the revised childhood anxiety sensitivity index (CASI-R) in a child and adolescent sample. *Journal of Psychopathology and Behavioral Assessment,**41*(4), 677–691. 10.1007/s10862-019-09745-y

[CR28] Francis, S. E., Noël, V. A., & Ryan, S. L. (2019b). A systematic review of the factor structure of anxiety sensitivity among children: Current status and recommendations for future directions. *Child & Youth Care Forum,**48*(5), 603–632. 10.1007/s10566-019-09502-y

[CR29] Franco, X., Saavedra, L. M., & Silverman, W. K. (2007). External validation of comorbid patterns of anxiety disorders in children and adolescents. *Journal of Anxiety Disorders,**21*(5), 717–729. 10.1016/j.janxdis.2006.10.002 17095184 10.1016/j.janxdis.2006.10.002 PMC2692683

[CR30] Garb, H. N. (2003). Incremental validity and the assessment of psychopathology in adults. *Psychological Assessment,**15*(4), 508–520. 10.1037/1040-3590.15.4.508 14692846 10.1037/1040-3590.15.4.508

[CR104] George, D., & Mallery, P. (2003). *SPSS for Windows step by step: A simple guide and reference*. 11.0 update. (4th ed.). Allyn & Bacon.

[CR105] George, D., & Mallery, P. (2010). *SPSS for Windows step by step: A simple guide and reference*. 17.0 Update. (10th ed.). Allyn & Bacon.

[CR31] Hallion, L. S., Hsu, K. J., & Schleider, J. L. (2024). Cognitive training for mental health problems. *Nature Mental Health,**2*(1), 17–24. 10.1038/s44220-023-00185-y

[CR32] Haynes, S. N., & Lench, H. C. (2003). Incremental validity of new clinical assessment measures. *Psychological Assessment,**15*(4), 456–466. 10.1037/1040-3590.15.4.456 14692842 10.1037/1040-3590.15.4.456

[CR33] Henderson, H. A. (2010). Electrophysiological correlates of cognitive control and the regulation of shyness in children. *Developmental Neuropsychology,**35*(2), 177–193. 10.1080/87565640903526538 20390601 10.1080/87565640903526538

[CR34] Hensley, L., & Varela, R. E. (2008). PTSD symptoms and somatic complaints following hurricane Katrina: The roles of trait anxiety and anxiety sensitivity. *Journal of Clinical Child & Adolescent Psychology,**37*(3), 542–552. 10.1080/15374410802148186 18645745 10.1080/15374410802148186

[CR35] Hernandez Rodriguez, J., Gregus, S. J., Craig, J. T., Pastrana, F. A., & Cavell, T. A. (2020). Anxiety sensitivity and children’s risk for both internalizing problems and peer victimization experiences. *Child Psychiatry & Human Development,**51*(2), 174–186. 10.1007/s10578-019-00919-z 31401756 10.1007/s10578-019-00919-z

[CR36] Hinshaw, S. P. (2017). Developmental Psychopathology as a Scientific Discipline. In *Child and Adolescent Psychopathology, Third Edition* (pp. 3–32). John Wiley & Sons, Ltd. 10.1002/9781394258932.ch1

[CR37] Isolan, L., Salum, G., Flores, S. M., de Carvalho, H. W., & Manfro, G. G. (2012). Reliability and convergent validity of the childhood anxiety sensitivity index in children and adolescents. *Jornal Brasileiro De Psiquiatria,**61*, 193–198. 10.1590/S0047-20852012000400001

[CR38] Jandrić, S., Filaković, P., Kurtović, A., Kovač, V., Benić, D., Rogulja, S., & Dodig-Ćurković, K. (2021). The role of cognitive control and rumination in predicting depression among adolescents with internalizing disorders. *Psychiatria Danubina,**33*(2), 165–172. 10.24869/psyd.2021.165 34185737 10.24869/psyd.2021.165

[CR39] Kalin, N. H. (2020). The critical relationship between anxiety and depression. *American Journal of Psychiatry,**177*(5), 365–367. 10.1176/appi.ajp.2020.20030305 32354270 10.1176/appi.ajp.2020.20030305

[CR40] Kim, J. H. (2019). Multicollinearity and misleading statistical results. *Korean Journal of Anesthesiology,**72*(6), 558–569. 10.4097/kja.19087 31304696 10.4097/kja.19087 PMC6900425

[CR41] Klein, A. M., Rapee, R. M., Hudson, J. L., Morris, T. M., Schneider, S. C., Schniering, C. A., Becker, E. S., & Rinck, M. (2019). Content-specific interpretation biases in clinically anxious children. *Behaviour Research and Therapy,**121*, 103452. 10.1016/j.brat.2019.103452 31430687 10.1016/j.brat.2019.103452

[CR42] Knapp, A. A., Feldner, M., Allan, N. P., Schmidt, N. B., Keough, M. E., & Leen-Feldner, E. W. (2020). Test of an anxiety sensitivity amelioration program for at-risk youth (ASAP-Y). *Behaviour Research and Therapy,**126*, 103544. 10.1016/j.brat.2019.103544 31981802 10.1016/j.brat.2019.103544 PMC7784583

[CR43] Kopala-Sibley, D. C., Olino, T., Durbin, E., Dyson, M. W., & Klein, D. N. (2018). The stability of temperament from early childhood to early adolescence: A multi-method. *Multi-Informant Examination. European Journal of Personality,**32*(2), 128–145. 10.1002/per.2151 30858648 10.1002/per.2151 PMC6407883

[CR44] Kozak, M. J., & Cuthbert, B. N. (2016). The NIMH research domain criteria initiative: Background, issues, and pragmatics. *Psychophysiology,**53*(3), 286–297. 10.1111/psyp.12518 26877115 10.1111/psyp.12518

[CR45] Lawson, K. M., Atherton, O. E., & Robins, R. W. (2021). The structure of adolescent temperament and associations with psychological functioning: A replication and extension of Snyder et al. (2105). *Journal of Personality and Social Psychology,**121*(5), e19–e39. 10.1037/pspp0000380 33539154 10.1037/pspp0000380 PMC8333190

[CR46] Leen-Feldner, E. W., Feldner, M. T., Bernstein, A., McCormick, J. T., & Zvolensky, M. J. (2005). Anxiety sensitivity and anxious responding to bodily sensations: A test among adolescents using a voluntary hyperventilation challenge. *Cognitive Therapy and Research,**29*(5), 593–609. 10.1007/s10608-005-3510-5

[CR47] Leen-Feldner, E. W., Feldner, M. T., Reardon, L. E., Babson, K. A., & Dixon, L. (2008). Anxiety sensitivity and posttraumatic stress among traumatic event-exposed youth. *Behaviour Research and Therapy,**46*(4), 548–556. 10.1016/j.brat.2008.01.014 18328463 10.1016/j.brat.2008.01.014 PMC2362392

[CR48] Leitenberg, H., Yost, L. W., & Carroll-Wilson, M. (1986). Negative cognitive errors in children: Questionnaire development, normative data, and comparisons between children with and without self-reported symptoms of depression, low self-esteem, and evaluation anxiety. *Journal of Consulting and Clinical Psychology,**54*(4), 528–536. 10.1037/0022-006X.54.4.528 3745607 10.1037//0022-006x.54.4.528

[CR49] Lovibond, P. F., & Lovibond, S. H. (1995). The structure of negative emotional states: Comparison of the depression anxiety stress scales (DASS) with the beck depression and anxiety inventories. *Behaviour Research and Therapy,**33*(3), 335–343. 10.1016/0005-7967(94)00075-u 7726811 10.1016/0005-7967(94)00075-u

[CR50] Luby, J. L. (2013). Treatment of anxiety and depression in the preschool period. *Journal of the American Academy of Child & Adolescent Psychiatry,**52*(4), 346–358. 10.1016/j.jaac.2013.01.011 23582866 10.1016/j.jaac.2013.01.011 PMC3640809

[CR51] Lunetti, C., Iselin, A.-M.R., Di Giunta, L., Lansford, J. E., Eisenberg, N., Pastorelli, C., Bacchini, D., Uribe Tirado, L. M., Thartori, E., Basili, E., Fiasconaro, I., Favini, A., Gerbino, M., Cirimele, F., Remondi, C., Skinner, A. T., & Rothenberg, W. A. (2022). Development of internalizing symptoms during adolescence in three countries: The role of temperament and parenting behaviors. *European Child & Adolescent Psychiatry,**31*(6), 947–957. 10.1007/s00787-021-01725-6 33547952 10.1007/s00787-021-01725-6 PMC8610087

[CR52] Maciejewski, D. F., van Lier, P. A. C., Branje, S. J. T., Meeus, W. H. J., & Koot, H. M. (2017). A daily diary study on adolescent emotional experiences: Measurement invariance and developmental trajectories. *Psychological Assessment,**29*(1), 35–49. 10.1037/pas0000312 27099977 10.1037/pas0000312

[CR53] MacNeill, L. A., & Pérez-Edgar, K. (2020). Temperament and Emotion. In *The Encyclopedia of Child and Adolescent Development* (pp. 1–12). John Wiley & Sons, Ltd. 10.1002/9781119171492.wecad180

[CR54] Marsee, M. A., Weems, C. F., & Taylor, L. K. (2008). Exploring the association between aggression and anxiety in youth: A look at aggressive subtypes, gender, and social cognition. *Journal of Child and Family Studies,**17*(1), 154–168. 10.1007/s10826-007-9154-1

[CR55] Martin, L., Kidd, M., & Seedat, S. (2016). Anxiety sensitivity in school attending youth: exploratory and confirmatory factor analysis of the 18-Item CASI in a multicultural South African sample. *Frontiers in Psychology*. 10.3389/fpsyg.2015.01996 26779098 10.3389/fpsyg.2015.01996 PMC4703811

[CR56] McDermott, J. M., Perez-Edgar, K., Henderson, H. A., Chronis-Tuscano, A., Pine, D. S., & Fox, N. A. (2009). A history of childhood behavioral inhibition and enhanced response monitoring in adolescence are linked to clinical anxiety. *Biological Psychiatry,**65*(5), 445–448. 10.1016/j.biopsych.2008.10.043 19108817 10.1016/j.biopsych.2008.10.043 PMC2788124

[CR57] Melton, T. H., Croarkin, P. E., Strawn, J. R., & McClintock, S. M. (2016). Comorbid anxiety and depressive symptoms in children and adolescents: A systematic review and analysis. *Journal of Psychiatric Practice,**22*(2), 84–98. 10.1097/PRA.0000000000000132 27138077 10.1097/PRA.0000000000000132 PMC6267783

[CR58] Micco, J. A., Henin, A., & Hirshfeld-Becker, D. R. (2014). Efficacy of interpretation bias modification in depressed adolescents and young adults. *Cognitive Therapy and Research,**38*(2), 89–102. 10.1007/s10608-013-9578-4 24729643 10.1007/s10608-013-9578-4 PMC3979637

[CR59] Miers, A. C., Blöte, A. W., Heyne, D. A., & Westenberg, P. M. (2014). Developmental pathways of social avoidance across adolescence: The role of social anxiety and negative cognition. *Journal of Anxiety Disorders,**28*(8), 787–794. 10.1016/j.janxdis.2014.09.008 25265547 10.1016/j.janxdis.2014.09.008

[CR60] Morales, S., Tang, A., Bowers, M. E., Miller, N. V., Buzzell, G. A., Smith, E., Seddio, K., Henderson, H. A., & Fox, N. A. (2022). Infant temperament prospectively predicts general psychopathology in childhood. *Development and Psychopathology,**34*(3), 774–783. 10.1017/S0954579420001996 33432897 10.1017/S0954579420001996 PMC8273182

[CR61] Morea, A., & Calvete, E. (2021). Cognitive flexibility and selective attention’s associations with internalizing symptoms in adolescents: Are they reciprocal? *Journal of Youth and Adolescence,**50*(5), 921–934. 10.1007/s10964-021-01402-6 33575916 10.1007/s10964-021-01402-6

[CR62] Naragon-Gainey, K. (2010). Meta-analysis of the relations of anxiety sensitivity to the depressive and anxiety disorders. *Psychological Bulletin,**136*(1), 128–150. 10.1037/a0018055 20063929 10.1037/a0018055

[CR63] Nivard, M. G., Lubke, G. H., Dolan, C. V., Evans, D. M., St Pourcain, B., Munafò, M. R., & Middeldorp, C. M. (2017). Joint developmental trajectories of internalizing and externalizing disorders between childhood and adolescence. *Development and Psychopathology,**29*(3), 919–928. 10.1017/S0954579416000572 27427290 10.1017/S0954579416000572

[CR64] Nye, A., Delgadillo, J., & Barkham, M. (2023). Efficacy of personalized psychological interventions: A systematic review and meta-analysis. *Journal of Consulting and Clinical Psychology,**91*(7), 389–397. 10.1037/ccp0000820 37166831 10.1037/ccp0000820

[CR65] Olthuis, J. V., Stewart, S. H., Watt, M. C., Sabourin, B. C., & Keogh, E. (2012). Anxiety sensitivity and negative interpretation biases: Their shared and unique associations with anxiety symptoms. *Journal of Psychopathology and Behavioral Assessment,**34*(3), 332–342. 10.1007/s10862-012-9286-5

[CR66] Pereira, A. I., Muris, P., Roberto, M. S., Marques, T., Goes, R., & Barros, L. (2018). Examining the mechanisms of therapeutic change in a cognitive-behavioral intervention for anxious children: The role of interpretation bias, perceived control, and coping strategies. *Child Psychiatry & Human Development,**49*(1), 73–85. 10.1007/s10578-017-0731-2 28500435 10.1007/s10578-017-0731-2

[CR67] Pérez-Edgar, K., Reeb-Sutherland, B. C., McDermott, J. M., White, L. K., Henderson, H. A., Degnan, K. A., Hane, A. A., Pine, D. S., & Fox, N. A. (2011). Attention biases to threat link behavioral inhibition to social withdrawal over time in very young children. *Journal of Abnormal Child Psychology,**39*(6), 885–895. 10.1007/s10802-011-9495-5 21318555 10.1007/s10802-011-9495-5 PMC3756613

[CR68] Pérez-Edgar, K., Taber-Thomas, B., Auday, E., & Morales, S. (2014). Temperament and attention as core mechanisms in the early emergence of anxiety. *Contributions to Human Development,**26*, 42. 10.1159/000354350 26663953 10.1159/000354350 PMC4671515

[CR69] Pössel, P., Seely, H. D., & Marchetti, I. (2024). Similarities and differences in the architecture of cognitive vulnerability to depressive symptoms in black and white American adolescents: A network analysis study. *Research on Child and Adolescent Psychopathology,**52*(10), 1591–1605. 10.1007/s10802-024-01218-5 38850463 10.1007/s10802-024-01218-5

[CR70] Prinstein, M. J., Cheah, C. S. L., & Guyer, A. E. (2005). Peer victimization, cue interpretation, and internalizing symptoms: Preliminary concurrent and longitudinal findings for children and adolescents. *Journal of Clinical Child & Adolescent Psychology,**34*(1), 11–24. 10.1207/s15374424jccp3401_2 15677277 10.1207/s15374424jccp3401_2

[CR71] Raines, E. M., Viana, A. G., Trent, E. S., Woodward, E. C., Candelari, A. E., Zvolensky, M. J., & Storch, E. A. (2019). Effortful control, interpretation biases, and child anxiety symptom severity in a sample of children with anxiety disorders. *Journal of Anxiety Disorders,**67*, 102136. 10.1016/j.janxdis.2019.102136 31494512 10.1016/j.janxdis.2019.102136 PMC6750729

[CR72] Reiss, S. (1991). Expectancy model of fear, anxiety, and panic. *Clinical Psychology Review,**11*(2), 141–153. 10.1016/0272-7358(91)90092-9

[CR73] Reiss, S., Peterson, R. A., Gursky, D. M., & McNally, R. J. (1986). Anxiety sensitivity, anxiety frequency and the prediction of fearfulness. *Behaviour Research and Therapy,**24*(1), 1–8. 10.1016/0005-7967(86)90143-9 3947307 10.1016/0005-7967(86)90143-9

[CR74] Reuland, M. M., & Teachman, B. A. (2014). Interpretation bias modification for youth and their parents: A novel treatment for early adolescent social anxiety. *Journal of Anxiety Disorders,**28*(8), 851–864. 10.1016/j.janxdis.2014.09.011 25445075 10.1016/j.janxdis.2014.09.011 PMC4303587

[CR75] Rozenman, M., Amir, N., & Weersing, V. R. (2014). Performance-based interpretation bias in clinically anxious youths: relationships with attention, anxiety, and negative cognition. *Behavior Therapy,**45*(5), 594–605. 10.1016/j.beth.2014.03.009 25022771 10.1016/j.beth.2014.03.009

[CR76] Sauer-Zavala, S., Wilner, J. G., & Barlow, D. H. (2017). Addressing neuroticism in psychological treatment. *Personality Disorders: Theory, Research, and Treatment,**8*(3), 191–198. 10.1037/per0000224 10.1037/per0000224 29120218

[CR77] Schwartz, J. S., & Maric, M. (2015). Negative cognitive errors in youth: Specificity to anxious and depressive symptoms and age differences. *Behavioural and Cognitive Psychotherapy,**43*(5), 526–537. 10.1017/S1352465814000228 26202072 10.1017/S1352465814000228

[CR78] Schweizer, T. H., Snyder, H. R., & Hankin, B. L. (2020). A reformulated architecture of cognitive risks for psychopathology: Common and specific dimensions and links to internalizing outcomes in adolescence. *Assessment,**27*(2), 334–355. 10.1177/1073191118804878 30295055 10.1177/1073191118804878 PMC6581618

[CR79] Sheehan, D. V., Lecrubier, Y., Sheehan, K. H., Amorim, P., Janavs, J., Weiller, E., Hergueta, T., Baker, R., & Dunbar, G. C. (1998). The Mini-International Neuropsychiatric Interview (M.I.N.I.): The development and validation of a structured diagnostic psychiatric interview for DSM-IV and ICD-10. *The Journal of Clinical Psychiatry*, *59 Suppl 20*, 22–33;quiz 34–57. 9881538

[CR80] Sheehan, D. V., Sheehan, K. H., Shytle, R. D., Janavs, J., Bannon, Y., Rogers, J. E., Milo, K. M., Stock, S. L., & Wilkinson, B. (2010). Reliability and validity of the mini international neuropsychiatric interview for children and adolescents (MINI-KID). *The Journal of Clinical Psychiatry,**71*(3), 313–326. 10.4088/JCP.09m05305whi 20331933 10.4088/JCP.09m05305whi

[CR81] Sherman, J. A., Braun, D. A., & Ehrenreich-May, J. (2019). 7—Anxiety sensitivity treatment for children and adolescents. In J. A. J. Smits, M. W. Otto, M. B. Powers, & S. O. Baird (Eds.), *The Clinician’s Guide to Anxiety Sensitivity Treatment and Assessment* (pp. 121–144). Academic Press. 10.1016/B978-0-12-813495-5.00007-3

[CR82] Shiner, R. L. (2015). The development of temperament and personality traits in childhood and adolescence. In *APA handbook of personality and social psychology, Volume 4: Personality processes and individual differences* (pp. 85–105). American Psychological Association. 10.1037/14343-004

[CR83] Silva, S. A., Silva, S. U., Ronca, D. B., Gonçalves, V. S. S., Dutra, E. S., & Carvalho, K. M. B. (2020). Common mental disorders prevalence in adolescents: A systematic review and meta-analyses. *PLoS ONE,**15*(4), e0232007. 10.1371/journal.pone.0232007 32324835 10.1371/journal.pone.0232007 PMC7179924

[CR84] Silverman, W. K., Fleisig, W., Rabian, B., & Peterson, R. A. (1991). Child anxiety sensitivity index. *Journal of Clinical Child Psychology,**20*(2), 162–168. 10.1207/s15374424jccp2002_7

[CR85] Silverman, W. K., Goedhart, A. W., Barrett, P., & Turner, C. (2003). The facets of anxiety sensitivity represented in the childhood anxiety sensitivity index: Confirmatory analyses of factor models from past studies. *Journal of Abnormal Psychology,**112*(3), 364. 10.1037/0021-843X.112.3.364 12943015 10.1037/0021-843x.112.3.364

[CR86] Smith, E. M., Reynolds, S., Orchard, F., Whalley, H. C., & Chan, S. W. (2018). Cognitive biases predict symptoms of depression, anxiety and wellbeing above and beyond neuroticism in adolescence. *Journal of Affective Disorders,**241*, 446–453. 10.1016/j.jad.2018.08.051 30145516 10.1016/j.jad.2018.08.051

[CR87] Smith, G. T., Fischer, S., & Fister, S. M. (2003). Incremental validity principles in test construction. *Psychological Assessment,**15*(4), 467–477. 10.1037/1040-3590.15.4.467 14692843 10.1037/1040-3590.15.4.467

[CR88] Snyder, H. R., Gulley, L. D., Bijttebier, P., Hartman, C. A., Oldehinkel, A. J., Mezulis, A., Young, J. F., & Hankin, B. L. (2015). Adolescent emotionality and effortful control: Core latent constructs and links to psychopathology and functioning. *Journal of Personality and Social Psychology,**109*(6), 1132–1149. 10.1037/pspp0000047 26011660 10.1037/pspp0000047 PMC4659771

[CR89] Stevanovic, D., Bagheri, Z., Atilola, O., Vostanis, P., Stupar, D., Moreira, P., Franic, T., Davidovic, N., Knez, R., Nikšić, A., Dodig-Ćurković, K., Avicenna, M., Noor, I. M., Nussbaum, L., Deljkovic, A., Thabet, A. A., Petrov, P., Ubalde, D., Monteiro, L. A., & Ribas, R. (2017). Cross-cultural measurement invariance of the revised child anxiety and depression scale across 11 world-wide societies. *Epidemiology and Psychiatric Sciences,**26*(4), 430–440. 10.1017/S204579601600038X 27353487 10.1017/S204579601600038X PMC6998552

[CR90] Stevanovic, D., Lalic, B., Batinic, J., Damjanovic, R., Jovic, V., Brkic-Cvetkovic, S., & Jancic, J. (2016). Children’s negative cognitive error questionnaire—revised: The factor structure and associations with anxiety and depressive symptoms across age, gender, and clinical/community samples. *Cognitive Therapy and Research,**40*(4), 584–592. 10.1007/s10608-016-9767-z

[CR91] Taber, K. S. (2018). The use of Cronbach’s alpha when developing and reporting research instruments in science education. *Research in Science Education,**48*(6), 1273–1296. 10.1007/s11165-016-9602-2

[CR92] Tairi, T., Adams, B., & Zilikis, N. (2016). Cognitive errors in greek adolescents: The linkages between negative cognitive errors and anxious and depressive symptoms. *International Journal of Cognitive Therapy,**9*(3), 261–278. 10.1521/ijct_2016_09_11

[CR93] Taylor, S., Zvolensky, M. J., Cox, B. J., Deacon, B., Heimberg, R. G., Ledley, D. R., Abramowitz, J. S., Holaway, R. M., Sandin, B., Stewart, S. H., Coles, M., Eng, W., Daly, E. S., Arrindell, W. A., Bouvard, M., & Cardenas, S. J. (2007). Robust dimensions of anxiety sensitivity: Development and initial validation of the Anxiety Sensitivity Index-3. *Psychological Assessment,**19*(2), 176–188. 10.1037/1040-3590.19.2.176 17563199 10.1037/1040-3590.19.2.176

[CR94] Trent, E. S., Viana, A. G., Raines, E. M., Woodward, E. C., Candelari, A. E., Storch, E. A., & Zvolensky, M. J. (2020). Interpretation biases and childhood anxiety: The moderating role of parasympathetic nervous system reactivity. *Journal of Abnormal Child Psychology,**48*(3), 419–433. 10.1007/s10802-019-00605-7 31802369 10.1007/s10802-019-00605-7

[CR95] Trent, L. R., Buchanan, E., Ebesutani, C., Ale, C. M., Heiden, L., Hight, T. L., Damon, J. D., & Young, J. (2013). A measurement invariance examination of the revised child anxiety and depression scale in a southern sample: Differential item functioning between African American and Caucasian youth. *Assessment,**20*(2), 175–187. 10.1177/1073191112450907 22855507 10.1177/1073191112450907

[CR96] Vergunst, F., Commisso, M., Geoffroy, M.-C., Temcheff, C., Poirier, M., Park, J., Vitaro, F., Tremblay, R., Côté, S., & Orri, M. (2023). Association of childhood externalizing, internalizing, and comorbid symptoms with long-term economic and social outcomes. *JAMA Network Open,**6*(1), e2249568. 10.1001/jamanetworkopen.2022.49568 36622675 10.1001/jamanetworkopen.2022.49568 PMC9856729

[CR97] Viana, A. G., & Gratz, K. L. (2012). The role of anxiety sensitivity, behavioral inhibition, and cognitive biases in anxiety symptoms: Structural equation modeling of direct and indirect pathways. *Journal of Clinical Psychology,**68*(10), 1122–1141. 10.1002/jclp.21890 22777955 10.1002/jclp.21890

[CR98] Viana, A. G., Kiel, E. J., Alfano, C. A., Dixon, L. J., & Palmer, C. A. (2017). The contribution of temperamental and cognitive factors to childhood anxiety disorder symptoms: A closer look at negative affect, behavioral inhibition, and anxiety sensitivity. *Journal of Child and Family Studies,**26*(1), 194–204. 10.1007/s10826-016-0553-z

[CR99] Vrijsen, J. N., Grafton, B., Koster, E. H. W., Lau, J., Wittekind, C. E., Bar-Haim, Y., Becker, E. S., Brotman, M. A., Joormann, J., Lazarov, A., MacLeod, C., Manning, V., Pettit, J. W., Rinck, M., Salemink, E., Woud, M. L., Hallion, L. S., & Wiers, R. W. (2024). Towards implementation of cognitive bias modification in mental health care: State of the science, best practices, and ways forward. *Behaviour Research and Therapy,**179*, 104557. 10.1016/j.brat.2024.104557 38797055 10.1016/j.brat.2024.104557

[CR100] Weems, C. F., & Watts, S. E. (2005). Cognitive Models of Childhood Anxiety. In *Anxiety disorder research* (pp. 205–232). Nova Science Publishers.

[CR101] Weems, C. F., Berman, S. L., Silverman, W. K., & Saavedra, L. M. (2001). Cognitive errors in youth with anxiety disorders: The linkages between negative cognitive errors and anxious symptoms. *Cognitive Therapy and Research,**25*(5), 559–575. 10.1023/A:1005505531527

[CR102] Weems, C. F., Hayward, C., Killen, J., & Taylor, C. B. (2002). A longitudinal investigation of anxiety sensitivity in adolescence. *Journal of Abnormal Psychology,**111*(3), 471–477. 12150423

[CR103] Zavos, H. M. S., Rijsdijk, F. V., Gregory, A. M., & Eley, T. C. (2010). Genetic influences on the cognitive biases associated with anxiety and depression symptoms in adolescents. *Journal of Affective Disorders,**124*(1), 45–53. 10.1016/j.jad.2009.10.030 19945751 10.1016/j.jad.2009.10.030

